# A disparity between physician attitudes and practice regarding hyperglycemia in pediatric intensive care units in the United States: a survey on actual practice habits

**DOI:** 10.1186/cc8865

**Published:** 2010-02-03

**Authors:** Catherine M Preissig, Mark R Rigby

**Affiliations:** 1 Medical Center of Central Georgia, Department of Pediatrics, Division of Pediatric Critical Care Medicine, 777 Hemlock Street, Macon, Georgia, 31201, USA; 2 Emory University School of Medicine, Children's Healthcare of Atlanta at Egleston, Department of Pediatrics, Division of Pediatric Critical Care, 1405 Clifton Rd, Atlanta, Georgia, 30322, USA

## Abstract

**Introduction:**

Hyperglycemia is common in critically ill patients and is associated with increased morbidity and mortality. Strict glycemic control improves outcomes in some adult populations and may have similar effects in children. While glycemic control has become standard care in adults, little is known regarding hyperglycemia management strategies used by pediatric critical care practitioners. We sought to assess both the beliefs and practice habits regarding glycemic control in pediatric intensive care units (ICUs) in the United States (US).

**Methods:**

We surveyed 30 US pediatric ICUs from January to May 2009. Surveys were conducted by phone between the investigators and participating centers and consisted of a 22-point questionnaire devised to assess physician perceptions and center-specific management strategies regarding glycemic control.

**Results:**

ICUs included a cross section of centers throughout the US. Fourteen out of 30 centers believe all critically ill hyperglycemic *adults *should be treated, while 3/30 believe all critically ill *children *should be treated. Twenty-nine of 30 believe *some *subsets of adults with hyperglycemia should be treated, while 20/30 believe *some *subsets of children should receive glycemic control. A total of 70%, 73%, 80%, 27%, and 40% of centers believe hyperglycemia adversely affects outcomes in cardiac, trauma, traumatic brain injury, general medical, and general surgical pediatric patients, respectively. However, only six centers use a standard, uniform approach to treat hyperglycemia at their institution. Sixty percent of centers believe *hypo*glycemia is more dangerous than *hyper*glycemia. Seventy percent listed fear of management-induced hypoglycemia as a barrier to glycemic control at their center.

**Conclusions:**

Considerable disparity exists between physician beliefs and actual practice habits regarding glycemic control among pediatric practitioners, with few centers reporting the use of any consistent standard approach to screening and management. Physicians wishing to practice glycemic control in their critically ill pediatric patients may want to consider adopting center-wide uniform approaches to improve safety and efficacy of treatment.

## Introduction

Hyperglycemia in critically ill patients occurs frequently, is associated with increased morbidity and mortality, and studies in adults suggest that tight glycemic control with insulin may improve outcomes [1-14]. Questions regarding safety and efficacy of this therapy, extent of outcome improvement, goal blood glucose (BG) range, and target patient population for treatment are of significant debate [15-18]. However, despite these unresolved issues several medical advisory committees recommend glycemic control as standard care in adults [19-22].

Studies regarding hyperglycemia and glycemic control in pediatrics are limited. Those available demonstrate that high BG is prevalent and independently associated with increased morbidity and mortality [5-14]. To date, a single randomized controlled trial to assess whether glycemic control improves outcomes in pediatric critical illness has been published. In this study, although tight glycemic control reduced morbidity and mortality, approximately 25% of patients receiving this management developed severe hypoglycemia [23]. Despite strong data favoring treatment and official recommendations to practice glycemic control in critically ill adults, there are no definitive studies or guidelines to help steer the practice in pediatric critical care.

Recent studies indicate that hyperglycemia is a significant concern among physicians caring for critically ill children and suggest that glycemic management is routinely performed [24,25]. Our group developed and published a protocol to identify and manage hyperglycemia in critically ill children and adopted this practice as routine care in our pediatric intensive care unit (ICU) [11,13]. From current literature, however, it is difficult to discern the breadth and extent of actual glycemic control efforts adopted by other pediatric centers. To better determine how physician attitudes towards glycemic control translate to actual practice we conducted a survey to assess the beliefs and practice habits regarding glycemic control in a cross section of pediatric ICUs in the United States.

## Materials and methods

We conducted a survey to ascertain glycemic control beliefs and practice habits at different pediatric critical care centers in the United States. Participating centers were chosen in an effort to include institutions of varying size, geographic location, acuity, practice model (open versus closed unit, private versus public), and type (medical, surgical, cardiac, mixed). Our pediatric ICU was not included in this survey. Request for participation was sent electronically to attending physicians (either Division Chiefs or other faculty) at different centers between January and May 2009. Surveys were conducted primarily by phone call between the investigators and participating attending physicians. Three centers chose to complete the survey electronically instead of by phone for convenience. One physician was chosen as the spokesperson to represent their institution. All participating individuals had the opportunity to review the survey with their coworkers and colleagues to ensure that their responses were representative of their center's beliefs and practices.

The survey comprised a 22-point questionnaire. Questions were developed to investigate the actual practice habits of intensivists regarding glycemic control in non-diabetic hyperglycemic critically ill children. Sections within the survey included questions specific to pediatric ICU demographic and descriptive data, individual perceptions and beliefs regarding glycemic control in critically ill children, individual and center-specific threshold for treatment, method of treatment (if applicable), and safety and efficacy of management at each center.

Statistical analysis was conducted using a software package (SPSS for Windows, version 13.0.1, Chicago, IL, USA). We determined the significance of differences in responses between pediatric ICU centers with χ^2 ^analysis (for categoric variables) and independent Student *t*-test (for continuous variables). A *P *value < 0.05 was considered statistically significant.

## Results

Of 40 centers queried, 30 pediatric ICUs agreed to participate in our survey, equating to a response rate of 75%. Ten centers either did not respond to our electronic request for participation or were not able to respond in a timely manner. All participating centers responded to all items on the questionnaire. Table 1 details demographic data and descriptions of the 30 participating pediatric ICUs. Centers included ICUs of varying size (based on number of beds), admissions per year, model (urban, suburban, rural), geographic region, number of ICU physicians, and type (medical, surgical, cardiac, mixed, open versus closed unit) (Table 1). Most of the centers (27/30) were affiliated with pediatric residency programs, and 67% (20/30) were affiliated with pediatric critical care fellowship programs. Almost all (29/30) participating sites were university-affiliated.

**Table 1:** Description of participating pediatric ICUs

	Number of ICUs (% of Total)
Total Number of ICUs Surveyed	30

ICU Model	

Urban	19 (63)
Suburban	6 (20)
Rural	5 (17)

Type of ICU	

Medical	3 (10)
Surgical	0 (0)
Cardiac	1 (3)
Mixed Medical/Surgical	10 (33)
Mixed Medical/Surgical/Cardiac	16 (54)
Open ICU	8 (27)
Closed ICU	22 (73)
Utilizes Pediatric ICU Fellows	20 (66)
Utilizes Pediatric Residents	27 (90)

Number of ICU Beds	

<12	6 (20)
12 to 30	13 (43)
>30	11 (37)

Number of Critical Care Physicians	

≤ 6	9 (30)
7 to 12	12 (40)
>12	9 (30)

Admissions Per Year	

<1,000	8 (26)
1,000 to 2,000	11 (37)
>2,000	11 (37)

Region	

Northeast	9 (30)
Southeast	10 (33)
West	3 (10)
Central	8 (27)

Table 2 describes pediatric center-specific beliefs regarding hyperglycemia and glycemic control in critically ill patients. Fourteen (47%) pediatric centers believe that *all *critically ill *adults *with hyperglycemia should undergo some form of glycemic control, whereas only 3/30 (10%) stated that *all *critically ill *children *with hyperglycemia should be treated (*P *< 0.05). Almost all centers (29/30, 97%) believe that at least *some *subsets of adults with hyperglycemia should be routinely treated, while 20/30 (67%) stated that at least some subsets of children with hyperglycemia should routinely receive glycemic control (Table 2). There was a non-uniform response when sites were questioned whether hyperglycemia contributed to poor outcome in select subsets of pediatric patients. While most believe that hyperglycemia adversely affects outcomes in cardiac (70%), trauma (73%), and traumatic brain injury (80%) patients, significantly fewer thought that there was an effect on outcomes in general medical (27%) and surgical (40%) patients (*P *< 0.05).

**Table 2:** Pediatric ICU beliefs regarding glycemic control

	All ICUs N = 30 (% of Total)	Small ICUs* N = 6 (% of Total)	Medium ICUs† N = 13 (% of Total)	Large ICUs± N = 11 (% of Total)
Believe the following patients should be *treated *for hyperglycemia				

All critically ill adults	14 (47)	5 (83)	5 (38)	4 (36)
Subsets of critically ill adults	29 (97)	6 (100)	12 (92)	11 (100)
				
All critically ill children	3 (10)	2 (33)	0 (0)	1 (9)
Subsets of critically ill children	20 (67)	5 (83)	9 (69)	6 (55)

Center's most unified belief regarding hyperglycemia in critically ill children (allowed to choose one)				

Most hyperglycemic children should be treated with insulin as this may improve outcome	3 (10)	0 (0)	2 (15)	1 (9)

Some subsets of children should be treated with insulin as this may improve outcome	20 (67)	4 (67)	8 (62)	9 (82)

Children may benefit from glycemic control, but until further studies are available this practice should be avoided	6 (20)	2 (33)	3 (23)	1 (9)

Children may benefit from glycemic control, but the risks outweigh the benefits	0 (0)	0 (0)	0 (0)	0 (0)

* <12 beds; † 12-30 beds; ± >30 beds

To determine if there was a difference in attitude or practice habits based on ICU size, we analyzed responses based on ICU capacity. Significantly more (83%, 5/6) small ICUs (<12 beds) stated that subsets of critically ill children with hyperglycemia should be treated compared to large ICUs (>30 beds), in which only 55% (6/11) believed so (*P *< 0.05).

In contrast to other reports, our survey assessed *actual *glycemic control practice habits in pediatric ICUs in the United States. Despite most centers reporting that they believe hyperglycemia worsens outcomes in many of their patients, and that at least some subsets of pediatric patients may benefit from glycemic control, only two (7%) centers reported that their facility uses a standard approach to screen for and treat hyperglycemia. In addition, four other centers (13%) reported that they do have a standard approach to manage hyperglycemia despite no regular approach for screening (Table 3). The vast majority of centers surveyed (80%) do *not *have a regular or agreed upon approach to glycemic control. Small centers (<12 beds) were more likely to have a standard protocol for hyperglycemic treatment compared to moderate (12 to 30 beds) and large (>30 beds) ICUs, 33%, 15%, and 18%, respectively. For centers that do employ a standard treatment approach, all (6/6) indicated they may use insulin infusions for glycemic control, while some also attempt to manage hyperglycemia using intermittent insulin (subcutaneous or intravenous) and/or modification of dextrose in fluids. Three of six centers that use a standard approach to treatment employ a written insulin infusion protocol.

**Table 3:** Pediatric ICU approach to hyperglycemia screening and management

Survey question	All ICUs N = 30 (% of Total)	Small ICUs* N = 6 (% of Total)	Medium ICUs† N = 13 (% of Total)	Large ICUs± N = 11 (% of Total)
Centers that have a standard approach to screen for and treat hyperglycemia	2 (7)	0 (0)	0 (0)	2 (18)

Centers that have a standard approach to hyperglycemia treatment only	6 (20)	2 (33)	2 (15)	2 (18)

Centers that have neither a standard approach to screening or treatment	24 (80)	4 (67)	11 (85)	9 (82)

Management for centers that do have a standard approach to treating hyperglycemia				
Insulin infusion	6	2	2	2
Intermittent insulin (IV push or subcutaneous)	1	1	0	0
Manipulation of dextrose in IV fluids	1	0	1	0

Estimate of hyperglycemic patients that receive glycemic management at your center is				
>90%	2	0	1	0
51 to 90%	4	0	1	3
26 to 50%	4	1	1	3
1 to 25%	20	5	10	5
No one in our group practices glycemic control on any patient	0	0	0	0

* <12 beds; † 12-30 beds; ± >30 beds

**Figure 1:**
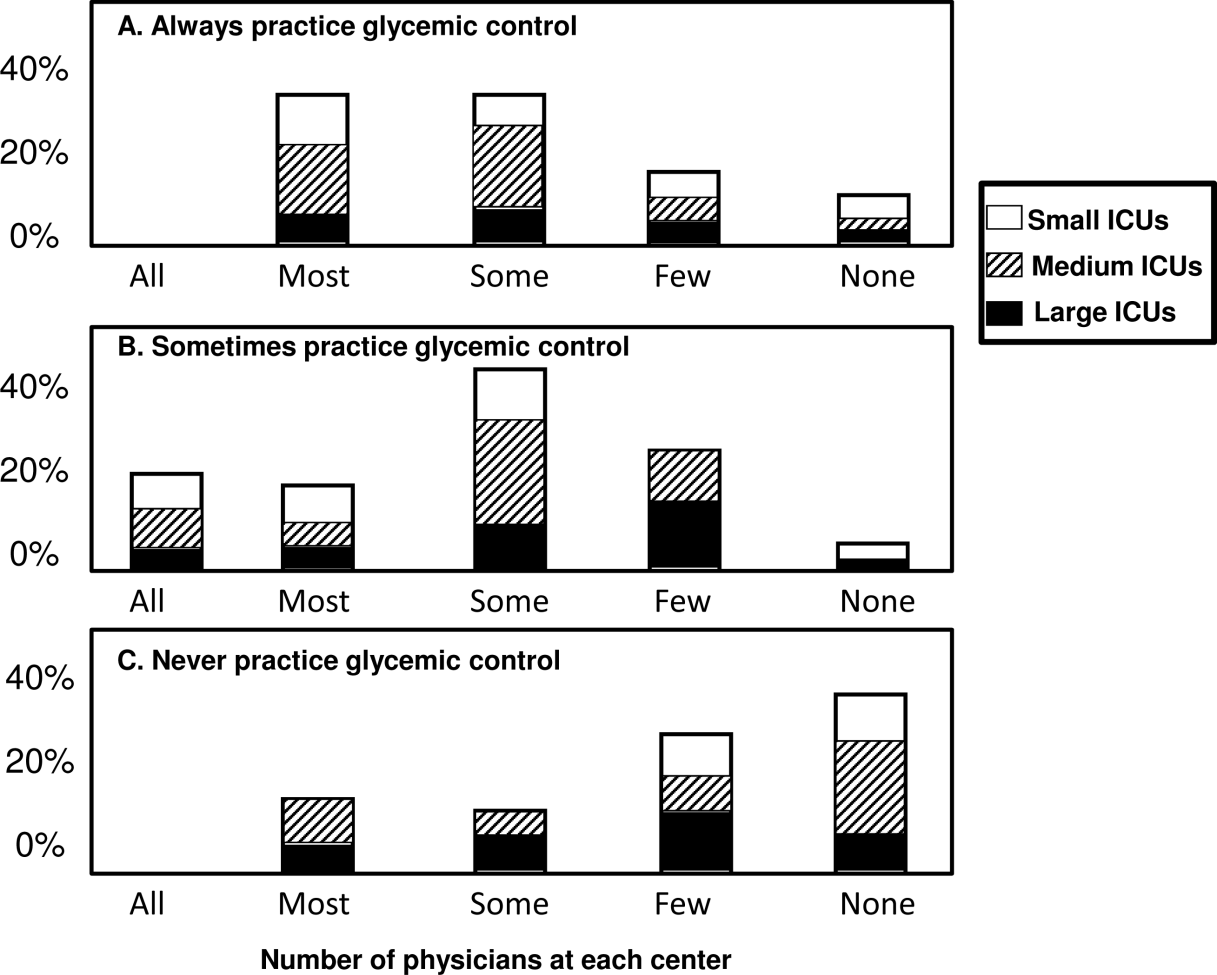
**Pediatric intensivist actual glycemic control practice habits**. Centers were queried regarding what percentage of practitioners always practice glycemic control, sometimes practice glycemic control, or never practice glycemic in all, most, some, few, and none of their hyperglycemic patients. Small ICU = <12 beds, Medium ICU = 12 to 30 beds, Large ICU = >30 beds. ICU = intensive care unit.

While few centers reported the use of any standard protocol for hyperglycemia management, we also assessed the use of glycemic control based on physician discretion at each center. When asked what percentage of hyperglycemic patients receive *any *treatment, either via a standard protocol used by all physicians or based on individual physician discretion, most centers (20/30, 67%) reported that likely only a minority (that is, 1 to 25%) of hyperglycemic patients receive *any *glycemic control. Figure 1 shows estimated numbers of physicians at each center that always, sometimes, or never treat critically ill children with hyperglycemia. Overall, no center reported that all of their physicians either always *or *never practice glycemic control. Approximately 35% of centers reported that most of their physicians always practice glycemic control, while 7% reported that most of their physicians never practice glycemic control. When broken down by ICU size, a proportionately higher number of small ICUs (<12 beds) were more likely to report that all or most of their physicians practice some type of glycemic control all or most of the time, and were more likely to report that few or none of their physicians never practice glycemic control (*P *< 0.05) (Figure 1). Half of the centers stated that for some of their physicians, the decision to treat hyperglycemia depended upon diagnosis, illness severity, and duration and severity of hyperglycemia. While most centers did not specify any agreed upon center-wide exclusions for glycemic management, three centers reported that they exclude infants and/or patients weighing <5 kg. Taken together, this data strongly indicate a large variation between glycemic control practices between pediatric ICUs, individual practitioners in any particular pediatric ICU, and at times even in the practice of any given physician.

**Figure 2:**
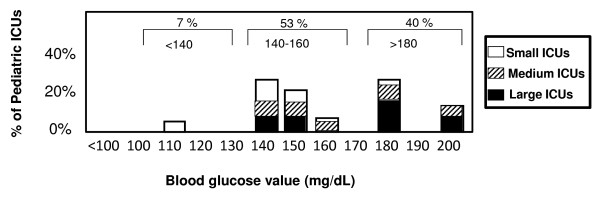
**Level of blood glucose to define hyperglycemia in different ICUs**. Centers were queried regarding their definition of hyperglycemia. Small ICU = <12 beds, Medium ICU = 12 to 30 beds, Large ICU = >30 beds. ICU = intensive care unit.

At present there is no consensus in critical care (adults or pediatrics) regarding the definition of *hyperglycemia *in critical illness. Figure 2 demonstrates that there is a wide variety of definitions of hyperglycemia employed at different pediatric centers. The BG above which pediatric critical care intensivists considered patients to be hyperglycemic ranged from 6 to 11 mmol/L (110 to 200 mg/dL), with most centers (>50%) defining a BG cut-off between 7.7 to 8.8 mmol/L (140 to 160 mg/dL). Large (>30 beds) ICUs were more likely to report a BG cut-off >9.9 mmol/L (180 mg/dL) (Figure 2). For physicians that do treat hyperglycemia, BG target ranges varied anywhere from a lower glucose limit of 3.8 mmol/L (70 mg/dL) to a maximum goal of 8.8 mmol/L (200 mg/dL). A goal range of 4.4 to 7.7 mmol/L (80 to 140 mg/dL) was the most consistent single target range reported (18/30 centers).

Centers were also asked what BG level they considered to be too low in critically ill children. The most common glucose level (47% centers) to define hypoglycemia was <2.2 mmol/L (40 mg/dL), followed by 37% of the centers using a BG <3.3 mmol/L (60 mg/dL), 10% using a BG of <4.4 mmol/L (80 mg/dL) and 3% using a cutoff of 2.8 mmol/L (50 mg/dL) or 5.5 mmol/L (100 mg/dL). Most centers (60%) believe that, in general, hypoglycemia is more dangerous than hyperglycemia. Although many centers have considered adopting a regular approach to glycemic management, 70% listed fear of management-induced hypoglycemia as a barrier to this practice in their unit.

## Discussion

For over three years our group has practiced glycemic control in our pediatric ICU as standard care. We routinely screen patients for hyperglycemia and implement a center-developed algorithm to maintain BG 4.4 to 7.7 mmol/L (80 to 140 mg/dL). We have previously defined the incidence and risk factors for hyperglycemia, and have demonstrated what appears to be an effective and safe approach to hyperglycemic management [11,13]. Despite recent debate regarding outcome improvements in adults and goal target glycemic ranges, numerous medical advisory groups recommend routine glycemic control as standard care in adult ICUs [19-22]. Because previous studies suggest most pediatric intensivists believe hyperglycemia may be hazardous to their patients, readers may infer that as in adult ICUs, glycemic control measures are the norm in pediatric ICU practice [24,25]. To ascertain the true practice patterns regarding glycemic control in critically ill children, we assessed beliefs and actual practice habits in a spectrum of pediatric ICUs in the United States.

Our survey suggests a considerable disparity between physician beliefs and actual practice habits among pediatric ICU practitioners, and is the first study to assess whether physician beliefs translate to practice strategies in pediatric ICUs in the United States. We find that beliefs and practice habits vary greatly between different centers, and even among practitioners from the same center. Recently a study from the United Kingdom also reported a wide variation of beliefs regarding glycemic control when respondents were queried about potential clinical scenarios [25].

The vast majority of adult ICUs have adopted regular approaches for glycemic control, and although the optimal goal BG target is unclear, there is little debate that glycemic control should be part of regular practice. Even following recent reports questioning outcome improvements and goal glycemic targets in adults, the American Diabetes Association, American College of Endocrinologist, and Institutes for Healthcare Improvements have all published recommendations that routine glycemic control be adopted in ICU-hospitalized adult patients [19-22]. It is of interest therefore that many of the respondents in our survey do not believe that all critically ill adults with hyperglycemia should undergo management, particularly as most pediatric ICUs do at times care for *adult *patients 18 to 21 years old.

Our study illuminates a dichotomy between pediatric ICU practitioner beliefs and practice. Although many pediatric intensivists believe hyperglycemia may worsen outcome and at least some subsets of patients may benefit from glycemic control, a significant minority of centers have implemented a routine approach to identify or treat hyperglycemia, as only 7% of centers reported a regular approach for hyperglycemia screening and management.

Admittedly there is little direct data indicating that glycemic control improves outcomes in critically ill children, yet a significant proportion of pediatric intensivists have apparently individually decided to incorporate glycemic control into practice while awaiting more definitive evidence. This has led to a wide variation in practice not only between centers, but frequently *within *the same practice group. This result raises concern on several levels. Although the particular glycemic metric for outcome improvement in adults with hyperglycemia is not clear, many reports suggest that in order to achieve clinical benefit, glycemic control must be maintained consistently throughout the ICU course [8,26,27]. During an ICU stay, one patient may be cared for by many providers, and if different triggers, therapeutic means, and targets for glycemic control of different providers are applied to a particular patient, any potential clinical benefit of glycemic control many be negated. In addition, disparate practice habits among members of the same physician group may lead to staff confusion and affect the success of glycemic management. Many centers that have been successful at instituting glycemic control measures find there is an important learning curve, and only with the proper education and experience can glycemic control measures be implemented effectively and safely [1-4,11,13]. Reducing practice variability and implementing methods to improve standardization of care have been important means to improve the quality of medical care delivered, reduce medical errors, and improve patient outcomes across the spectrum of medical disciplines. As such, even in the absence of direct evidence of improved outcomes with glycemic control in pediatric critical care, there may be good reason for pediatric groups interested in providing glycemic control to their patients to consider developing consistent, agreed-upon approaches to glycemic management in their ICUs.

This study also highlights some notable differences regarding hyperglycemia beliefs and practice strategies and ICU size. We found that smaller pediatric ICUs, that is, those with fewer ICU beds, annual admissions, and number of attending physicians, were more likely to treat hyperglycemia than larger institutions. Small ICUs rarely reported that no or few intensivists treat hyperglycemia, and many reported that most physicians do employ glycemic control most of the time. Proportionately, smaller ICUs were more likely to have adopted a standard approach to hyperglycemia management as well. Further, smaller ICUs believe glycemic control should be instituted at a lower BG threshold compared to larger ICUs, and were more likely to report a lower BG definition for hypoglycemia. Previous studies have not reviewed or mentioned similar discrepancies, but these differences may likely be due to the less challenging nature of devising and agreeing upon practice policies in smaller groups compared to those with many practitioners.

Similar to findings by others, we report that most pediatric ICU practitioners (60%) believe that *hypo*glycemia is more dangerous than *hyper*glycemia in critically ill children [24,25]. Although there are reports of immediate and long-term sequela from hypoglycemic episodes in children, the direct relationship of the severity and duration of hypoglycemia to adverse effects is unclear. The relatively recent influx of data showing high incidence, severity and correlation, and perhaps causal relationship of hyperglycemia with adverse effects in critical illness may begin to challenge practitioners' concepts of whether hypo or hyperglycemia is more detrimental. We found that 70% of centers reported that fear of iatrogenic hypoglycemia is a major, if not the primary, barrier to instituting routine glycemic control in their pediatric ICU. Indeed, studies in adult ICUs regarding glycemic control report hypoglycemic (BG <2.2 mmol/L, 40 mg/dL) rates as high as 40% in patients receiving tight control with insulin [3,26,27]. In addition, 25% of patients participating in the recent pediatric randomized controlled trial conducted in Belgium suffered from BG <2.2 mmol/L (40 mg/dL) [23]. These high profile reports likely will further contribute to fear and refractoriness of glycemic control in pediatric critical care. Yet there are numerous reports of adult centers that have implemented glycemic control measures without high incidence of hypoglycemia. Our own studies indicate that glycemic control can be implemented in pediatric medical/surgical and cardiac ICUs with little to no increase in hypoglycemic episodes [11,13]. Therefore elevated rates of iatrogenic hypoglycemia do not always necessarily follow the implementation of glycemic control protocols. Groups considering implementing glycemic control should realize that physician and staff education, training, and dedication may allow for the effective adoption of safe approaches to glycemic control.

Limitations of our study should be noted. While we attempted to target centers of varying size, geographic location, acuity, practice model, and type, data obtained from this survey only represents a portion of pediatric critical care centers nationally. However, as there are approximately 340 pediatric critical care centers in the United States, our survey of 30 centers does represent approximately 9 to 10% of all centers, and thus we believe does include a respectable sample size of pediatric institutions [28]. In addition, we only surveyed one individual from each pediatric center, as we were unable to include every physician at every institution in our evaluation. However, each individual chosen to represent their group for this study had the opportunity to discuss our survey questions with other members of their group to ensure responses adequately reflected those of their center.

Lastly, it is notable that results from at least two important studies in this field were published during the time this survey was conducted, specifically the aforementioned pediatric glycemic control trial by Vlasselaers *et al*, and more recently the results from the NICE-SUGAR investigators [15,23]. These studies potentially may have influenced current practice habits in participating centers. Findings from these studies add to the debate and controversy regarding strict versus conventional glycemic control, outcome improvements, and goal target BG levels in adult and pediatric populations. It is important to recognize that results from our survey represent a snap-shot of current trends in pediatric glycemic control, and that in this ever-evolving field, beliefs and practices will likely continue to change as more data becomes available to guide evidence-based practice.

## Conclusions

In summary, we find that there exists a significant awareness of hyperglycemia in pediatric ICU practice, but that few have modified their group practice to reflect their current beliefs. In general, pediatric intensivists may benefit from revisiting and staying abreast of the current state of literature regarding both hyper and hypoglycemia in critically ill children, and we recommend that all pediatric practitioners should consider treating hyperglycemia in their older, adult patients, such as those >18 years old, as suggested by multiple medical advisory groups. It may be premature to recommend the widespread adoption of glycemic control measures in all critically ill children on the basis of outcome studies, but for those centers that do practice glycemic control, there may be other quality and safety reasons to develop a center-consistent approach to this management. Support and encouragement of future studies to develop and validate safe and effective pediatric-specific approaches to glycemic control, and to assess whether this management impacts outcomes in critically ill children will be of utmost importance.

## Key messages

• Hyperglycemia is common in critically ill patients, is associated with increased morbidity and mortality, and strict glycemic control with insulin may improve outcomes in some populations.

• Most adult institutions have adopted regular approaches for glycemic control, and although the optimal goal BG target is unclear, many medical advisory committees recommend that at least some degree of glycemic control should be part of regular practice.

• There is a paucity of direct evidence for glycemic control in children; however, the only randomized glycemic control trial conducted in critically ill children to date does suggest outcome improvement with this therapy in this population.

• While most pediatric practitioners do believe hyperglycemia worsens outcomes in many of their patients, very few centers use a standard approach to treat hyperglycemia, and most that do attempt glycemic control use inconsistent, non-validated approaches.

• Recommendations for routine glycemic control in all pediatric ICU patients may be premature at this time, but pediatric centers wishing to practice glycemic control in their patients based on the most recent literature and studies suggesting potential outcome improvement may benefit from adopting a routine, center-consistent approach at their institution to optimize effectiveness and safety of this therapy.

## Abbreviations

BG: blood glucose; ICU: intensive care unit.

## Competing interests

The authors declare that they have no competing interests.

## Authors' contributions

Both authors of this manuscript contributed significantly and equally to this study, including study design, survey development, conduction of surveys, data gathering and analysis, and formal writing of this manuscript. All authors read and approved the final manuscript.
